# Impact of subchorionic hematoma in early pregnancy on obstetric complications: A retrospective cohort study in women who had live births after frozen‐thawed embryo transfer

**DOI:** 10.1002/rmb2.12343

**Published:** 2020-08-05

**Authors:** Shuhei So, Osamu Mochizuki, Wakasa Yamaguchi, Nao Murabayashi, Naomi Miyano, Fumiko Tawara

**Affiliations:** ^1^ Department of Reproductive and Perinatal Medicine Hamamatsu University School of Medicine Shizuoka Japan; ^2^ Tawara IVF Clinic Shizuoka Japan

**Keywords:** abnormal placental adhesion, assisted reproductive technology, pregnancy complications, subchorionic hematoma, vaginal bleeding

## Abstract

**Purpose:**

We investigated the contribution of subchorionic hematoma (SCH) involvement in early pregnancy to the risk of pregnancy complications in women who underwent frozen‐thawed embryo transfer (FET).

**Methods:**

A hypoechogenic area surrounding the gestational sac at early pregnancy on ultrasound was defined as SCH. Simultaneously, the presence of vaginal bleeding was evaluated. We included 1416 women with live births after FET between March 2015 and September 2018 in this study. The frequency of pregnancy complications was compared between the SCH (n = 340) and non‐SCH (n = 1076) groups.

**Results:**

The adjusted odds ratio of abnormal placental adhesion and placenta previa for the SCH group relative to the non‐SCH group was 7.01 [2.96‐18.00] and 3.77 [1.24‐11.91], respectively. In contrast, hypertensive disorders of pregnancy, non‐reassuring fetal status, fetal growth restriction, chorioamnionitis, and premature rupture of the membrane showed no differences between both groups. Furthermore, the frequency of abnormal placental adhesion was higher in the SCH group with vaginal bleeding than in the SCH group without vaginal bleeding.

**Conclusions:**

Subchorionic hematoma in early pregnancy may cause abnormal placental adhesion and placenta previa in pregnant women with FET. SCH presence should be carefully noted, particularly in cases with vaginal bleeding during early pregnancy after FET.

## Abstract 2

Subchorionic hematoma in early pregnancy may cause abnormal placental adhesion and placenta previa in pregnant women with frozen embryo transfer. SCH presence should be carefully noted, particularly in cases with vaginal bleeding during early pregnancy after frozen embryo transfer.

## 1 INTRODUCTION

In Japan, increasing freeze all strategies are employed to improve pregnancy rates and avoid ovarian hyperstimulation syndrome (OHSS).^1^, ^2^, ^3^, ^4^ However, some pregnancy complications are reportedly higher with frozen‐thawed embryo transfer (FET) than with fresh embryo transfer.^5^, ^6^ In FET, there are two main methods of endometrial preparation. One is the hormone replacement cycle (HRC) that regulates the endometrium with exogenous hormones before embryo transfer. Another is the natural cycle (NC) wherein the transfer is performed according to natural ovulation timing. To date, HRC is reportedly associated with a higher frequency of hypertensive disorders of pregnancy (HDP) and adherent placenta and a lower frequency of gestational diabetes (GDM) than is NC.^7^ However, it remains unknown why differences in endometrial preparation methods affect the frequency of pregnancy complications.

Subchorionic hematoma (SCH) is defined as a hypoechogenic area surrounding the gestational sac observed on ultrasound and contributes to the risks of miscarriage, preterm birth, and other pregnancy complications.^8^, ^9^, ^10^ Moreover, a higher incidence of SCH in pregnancy achieved by assisted reproductive technology (ART) than in spontaneous pregnancy has been reported.^11^ Therefore, the association of SCH in early pregnancy with ART‐related pregnancy complications is an interesting topic.

This study retrospectively investigated whether SCH in early pregnancy is involved in the development of pregnancy complications in women who underwent FET.

## 2 METHODS

### 2.1 Study population

**Figure 1:**
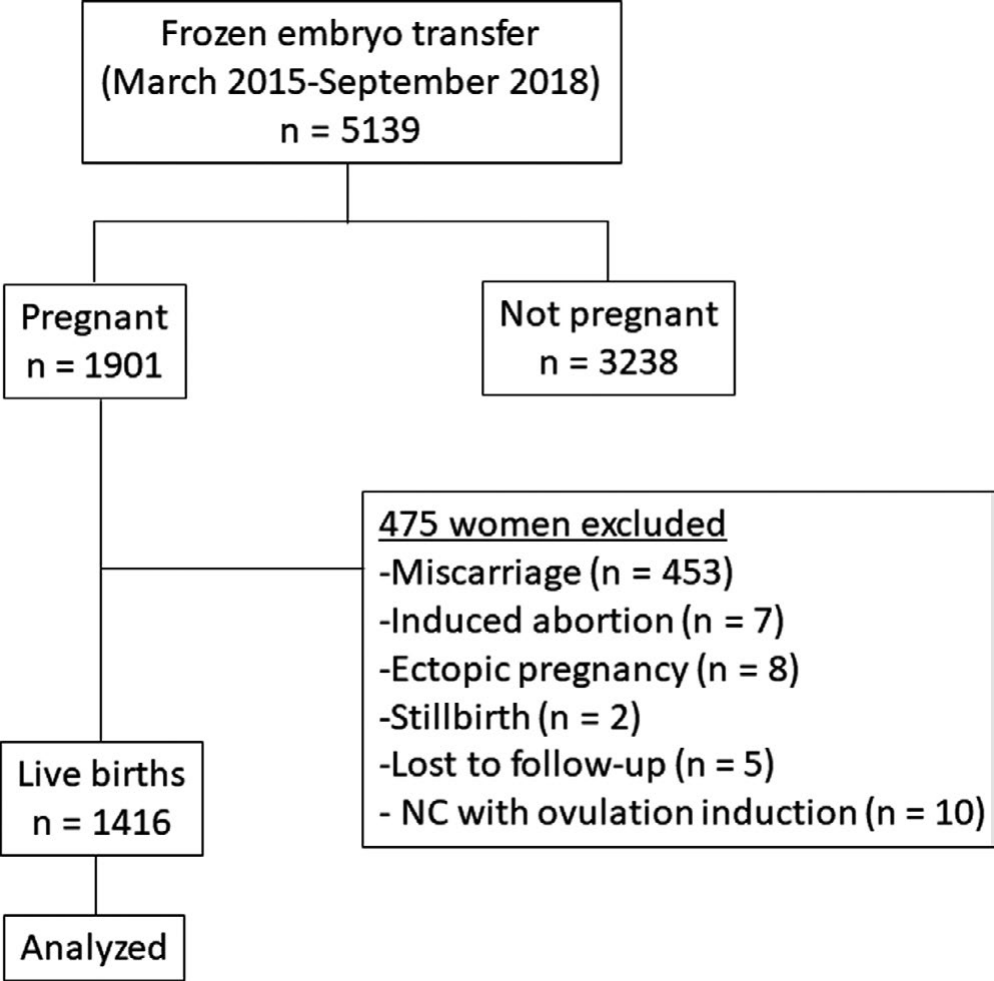
Flow diagram of study subjects

This study was approved by the Ethics Committee of Tawara IVF Clinic. Following the recruitment of study participants based on an opt‐out method, clinical information was collected via a review of their medical records. Of the 5139 patients who underwent FET between March 2015 and September 2018, 1901 were pregnant. Of these, the following cases were excluded: miscarriage (n = 453), induced abortion (n = 7), ectopic pregnancy (n = 8), stillbirth (n = 2), lost to follow‐up, including no echo examination at early pregnancy (n = 5), and NC with ovulation induction agents (n = 10). Overall, 1416 women who achieved live birth were included in this study (Figure 1). SCH was defined sonographically as a hypoechogenic area surrounding the gestational sac at 9 weeks of gestation or less. Simultaneously, the presence or absence of vaginal bleeding was evaluated. Abnormal placental adhesion was defined as a case that required the manual removal of the placenta or developed placental tissue defects. Obstetric complications data were obtained from delivery hospital records.

### 2.2 Endometrial preparation

Two methods of endometrial preparation were used in this study, namely the NC in patients with regular ovulatory cycles, including human chorionic gonadotropin trigger and HRC mainly for women without regular ovulatory cycles. In the HRC group, transdermal estradiol (Estrana tape^®^, Hisamitsu) was initiated on days 2‐4 of menstruation (2.26 mg/every 2 days) until 7 weeks of gestation. For luteal phase support, oral dydrogesterone (15 mg/day: Duphaston^®^, Solvay Pharma) was administered in both HRC and NC groups (up to 9 weeks of gestation for HRC and up to 7 weeks of gestation for NC). Furthermore, the recommended doses of vaginal progesterone were administered in the HRC group (up to 9 weeks of gestation); however, none or small amounts of that were administered in the NC group (up to 4 weeks of gestation).

### 2.3 Statistical analysis

Results were analyzed using t tests to compare both groups. The Pearson's chi‐square or Fisher's exact test was used depending on the number of observations in the table cells for comparison of proportion. Odds ratio (OR) and 95% confidence interval (CI) were calculated in the univariable and multivariable analyses. P value < 0.05 was considered statistically significant. The statistical analysis was performed using the R software and JMP9 software (SAS).

## 3 RESULTS

### 3.1 Subchorionic hematoma rate and patient background

Of the 1416 women, 340 (24.0%) developed SCH in early pregnancy. Table 1 shows a comparison of the patient backgrounds, including fertility treatment and the cause of infertility between the SCH group (n = 340) and the non‐SCH group (n = 1076). The SCH group had a lower BMI than did the non‐SCH group, with more histories of pregnancy. No difference was observed in age, history of miscarriage, and history of embryo transfer between the two groups. There was no difference between both groups in the methods of fertility treatment, including endometrial preparation methods, the number of transfer embryo, embryo stage at transfer, low‐dose aspirin during pregnancy, and endometrial thickness during the embryo transfer cycle. In terms of the causes of infertility, the SCH group had a significantly higher frequency of endometrial polyp and tubal factor than did the non‐SCH group. There was no between‐group difference in the frequency of dilatation and curettage (D&C), transcervical resection (TCR), and observation (as the treatment of endometrial polyps before embryo transfer) (Supplemental Table S1).

**Table 1:** Patient characteristics in the SCH and non‐SCH groups

	SCH (EFS) n = 340	Non‐SCH n = 1076	*P* value
Age^a^	35.2 ± 3.8	35.3 ± 4.0	.6446
BMI^a^	20.4 ± 2.7	20.9 ± 3.1	.0212
History of pregnancy^a^	0.82 ± 0.99	0.67 ± 0.93	.0161
History of miscarriage^a^	0.37 ± 0.63	0.32 ± 0.62	.1783
‐% ≥2	7.4%	5.7%	.2967
History of live birth^a^	0.28 ± 0.50	0.23 ± 0.45	.0622
History of embryo transfer^a^	4.17 ± 2.71	3.83 ± 2.44	.9111
Endometrial preparation methods			
HRC	62.6%	61.3%	.7012
NC	37.4%	38.7%	
No. of transfer embryo			
1	91.8%	94.3%	.0893
2	8.2%	5.7%	
Embryo stage at transfer			
Early embryo	24.4%	22.0%	.4798
Blastocyst	72.1%	75.2%	
2 step ET	3.5%	2.8%	
Good BT^b^	89.1%	87.9%	.6491
Low‐dose Aspirin during pregnancy	7.6%	9.5%	.3304
Endometrial thickness (mm) ^a^	9.9 ± 1.8	10.0 ± 1.7	.5954
Cause of infertility			
Male infertility	19.4%	21.7%	.4023
Ovulation factors	5.6%	4.9%	.6709
PCOS	4.4%	4.4%	1.0000
Uterine factors	17.1%	11.2%	.0065
Leiomyoma	5.6%	4.6%	.4668
Endometrial polyps	9.4%	4.9%	.0037
Adenomyosis	2.9%	2.2%	.4233
Tubal factor	15.0%	10.9%	.0435
Endometriosis	4.4%	4.4%	.4705

Abbreviations: BMI, body mass index; BT, blastocyst; EFS, echo free space; ET, embryo transfer; HRC, hormone replacement cycle; NC, natural ovulatory cycle; PCOS, polycystic ovarian syndrome; SCH, subchorionic hematoma.

^a^ Values are presented as mean ± SD.

^b^ Per single blastocyst transfer.

### 3.2 Delivery outcomes and pregnancy complications

No differences in gestational age, cesarean section rate, sex of offspring, birthweight, and preterm birth were observed between the SCH group and the non‐SCH group (Table 2). Although the SCH group demonstrated a higher frequency of abnormal placental adhesion than did the non‐SCH group (*P* < .0001), HDP, non‐reassuring fetal status (NRFS), fetal growth restriction (FGR), chorioamnionitis (CAM), premature rupture of the membranes (PROM), and placenta previa showed no differences between both groups (Tables 2). On the contrary, the results indicated that SCH was associated with placenta previa (AOR 3.77 [1.24‐11.91]) and abnormal placental adhesion (AOR 7.01 [2.96‐18.00]) after adjusted multivariate analysis (Table 3).

**Table 2:** Pregnancy outcomes in the SCH and non‐SCH groups

	SCH (EFS) n = 340	Non‐SCH n = 1076	*P* value
Obstetrical outcomes of live birth			
Gestational age at birth			
≤36 weeks	10.9% (37)	8.7% (94)	.4216
37‐41 weeks	85.6% (291)	88.7% (954)	
≥42 weeks	0.3% (1)	0.1% (1)	
Unknown	3.2% (11)	2.5% (27)	
Mode of delivery			
Vaginal delivery	55.0% (187)	58.6% (630)	.4777
Cesarean section	43.5% (148)	40.3% (434)	
Unknown	1.5% (5)	1.1% (12)	
Uterine Bleeding at Delivery (mL)^a^			
Vaginal delivery	778 ± 685	609 ± 521	.0015
Cesarean section	1066 ± 892	947 ± 622	.1499
Obstetrical complications			
HDP	8.5% (29)	8.6% (92)	1.000
NRFS	9.1% (31)	8.6% (92)	.7410
FGR	2.9% (10)	2.0% (21)	.2890
CAM	2.4% (8)	1.6% (17)	.3479
PROM	9.4% (32)	9.9% (106)	.9165
Placenta previa	3.5% (12)	1.9% (20)	.0915
Abnormal placental adhesion	9.4% (32)	3.0% (32)	<.0001
Sex of offspring ^a^			
Male	52.9% (175)	49.6% (523)	.2433
Female	45.3% (150)	47.0% (496)	
Unknown	3.4% (6)	6.9% (36)	
Birth weight^b^			
<2500 g	4.5% (13)	6.0% (57)	.0576
2500 g‐3999 g	94.4% (271)	90.2% (856)	
≥4000 g	0.7% (2)	0.9% (9)	
Unknown	0.3% (1)	2.8% (27)	

Abbreviations: CAM, chorioamnionitis; EFS, echo free space; FGR, fetal growth restriction; HDP, hypertensive disorders of pregnancy; NRFS, non‐reassuring fetal status; PROM, premature rupture of the membrane; SCH, subchorionic hematoma.

^a^ Per singleton.

^b^ Per singleton and term delivery.

**Table 3:** Crude and adjusted odds ratio of SCH against non‐SCH women for obstetrical outcomes

Outcomes	Crude OR (95% CI)	Adjusted OR (95% CI)	
Cesarean section	1.15 (0.90‐1.47)	1.05 (0.74‐1.49)	
Preterm delivery	1.29 (0.85‐1.91)	1.18 (0.65‐2.08)	
Obstetrical complications		
HDP	1.00 (0.66‐1.58)	0.94 (0.51‐1.66)	
NRFS	1.07 (0.69‐1.63)	1.62 (0.90‐2.84)	
FGR	1.52 (0.68‐3.19)	1.65 (0.53‐4.71)	
CAM	1.50 (0.61‐3.41)	2.17 (0.58‐7.62)	
PROM	0.95 (0.62‐1.43)	1.13 (0.64‐1.93)	
Placenta previa	1.93 (0.91‐3.94)	3.77 (1.24‐11.91)	
Abnormal placental adhesion	3.39 (2.04‐5.64)	7.01 (2.96‐18.00)	

Note

Adjusted for maternal age, BMI, history of pregnancy and miscarriage, embryo stage at transfer, number of the embryos transferred, use of assisted hatching, endometrial preparation method, Aspirin use during pregnancy, cause of infertility (male infertility, PCOS, leiomyoma, polyps, adenomyosis, fallopian tube obstruction, and endometriosis).

Abbreviations: CAM, chorioamnionitis; CI, confidence interval; FGR, fetal growth restriction; HDP, hypertensive disorders of pregnancy; NRFS, non‐reassuring fetal status; OR, odd ratio; PROM, premature rupture of the membrane; SCH, subchorionic hematoma.

Vaginal bleeding is a common characteristic of SCH, and the presence of vaginal bleeding has been reported to aggravate the state of complications in pregnant women with SCH.^12^ In this study, 46.8% (159/340) of patients with SCH experienced vaginal bleeding in early pregnancy. Therefore, this study investigated the influence of the presence of vaginal bleeding on the development of abnormal placental adhesion in the SCH group. Consequently, among patients who developed SCH, vaginal bleeding in the early stage of pregnancy was more frequently observed in the abnormal placental adhesion group than in the non‐abnormal placental adhesion group (75.0% vs 43.8%, *P* = .001). After adjusting for confounding factors, including patient backgrounds and fertile treatment methods, the prevalence of abnormal placental adhesion was significantly higher in the SCH group with vaginal bleeding than in the SCH group without vaginal bleeding (AOR, 4.52 [1.35‐17.62]). On the contrary, cesarean section, preterm delivery, HDP, NRFS, FGR, CAM, PROM, and placenta previa showed no significant difference between both groups (Table 4). Thus, SCH may be a risk factor for both abnormal placental adhesion and placenta previa; in addition, the risk of abnormal placental adhesion was significantly higher in cases of SCH with vaginal bleeding than in those without vaginal bleeding.

**Table 4:** Crude and adjusted odds ratio of SCH with vaginal bleeding against SCH without vaginal bleeding women for obstetrical outcomes

Outcomes	Crude OR (95% CI)	Adjusted OR (95% CI)	
Cesarean section	1.16 (0.75‐1.79)	0.97 (0.47‐2.01)	
Preterm delivery	1.55 (0.78‐3.13)	0.58 (0.13‐2.20)	
Obstetrical complications		
HDP	1.07 (0.49‐2.30)	1.60 (0.45‐5.63)	
NRFS	1.07 (0.51‐2.26)	1.01 (0.32‐3.03)	
FGR	0.75 (0.19‐2.68)	1.24 (0.13‐9.01)	
CAM	0.68 (0.14‐2.80)	0.55 (0.01‐7.36)	
PROM	1.15 (0.55‐2.41)	0.76 (0.23‐2.27)	
Placenta previa	0.56 (0.15‐1.81)	0.41 (0.03‐3.57)	
Abnormal placental adhesion	3.84 (1.74‐9.39)	4.52 (1.35‐17.62)	

Note

Adjusted for maternal age, BMI, history of pregnancy and miscarriage, embryo stage at transfer, number of the embryos transferred, use of assisted hatching, endometrial preparation method, Aspirin use during pregnancy, cause of infertility (male infertility, PCOS, leiomyoma, polyps, adenomyosis, fallopian tube obstruction, endometriosis).

Abbreviations: CAM, chorioamnionitis; CI, confidence interval; FGR, fetal growth restriction; HDP, hypertensive disorders of pregnancy; NRFS, non‐reassuring fetal status; OR, odd ratio; PROM, premature rupture of the membrane; SCH, subchorionic hematoma.

## 4 DISCUSSION

In this study, the proportions of patients with a history of pregnancy and endometrial polyp were significantly higher in the SCH group than in the non‐SCH group. More than half of patients with endometrial polyps had received D&C or TCR prior to the embryo transfer cycle. These results suggest that a history of intrauterine manipulations may affect SCH formation. Furthermore, we observed that tubal factor infertility was also frequent in the SCH group. The problem of the fallopian tubes was diagnosed as either unilateral or bilateral tubal block using hysterosalpingography. Such tubal adhesions and occlusions have been suggested to be triggered by bacteria‐induced inflammatory reactions.^13^ Therefore, it is possible that the inflammation of the fallopian tubes or uterus is one of the causes of SCH. Moreover, inflammatory cells express matrix metalloproteases (MMPs) that regulate trophoblast invasion.^14^, ^15^ Therefore, inappropriate expression of MMPs in patients with tubal factor infertility can be responsible for the development of SCH.

In the retrospective examination of the association between SCH in early pregnancy and pregnancy complications, it was observed that placenta previa and abnormal placental adhesion were more frequent in the SCH group than in the non‐SCH group. Abnormal placental adhesion can be accompanied by uterine bleeding due to placental separation. We observed that uterine bleeding at vaginal delivery was significantly higher in the SCH group than in the non‐SCH group. This result is in line with the observation that the frequency of abnormal placental adhesion is higher in the SCH group than in the non‐SCH group.

As an endometrial preparation, Saito et al previously reported that placental adhesion is more frequent in HRC than in NC.^7^ Similarly, the frequency of abnormal placental adhesion was significantly higher in HRC than in NC (data not shown) in this study. However, no significant difference in the frequency of SCH was observed between HRC and NC. Therefore, endometrial preparation by HRC and the development of SCH in early pregnancy may be independent risk factors for abnormal placental adhesion. Furthermore, although Truong et al reported that taking aspirin during pregnancy increases the development of SCH,^16^ no significant difference in aspirin use during pregnancy was observed between the SCH and non‐SCH groups in this study.

Nagy et al observed that SCH in the first trimester was associated with cesarean section, PIH, preeclampsia, placental abruption, abnormally adherent placentation, preterm delivery, FGR, and NRFS in a prospective study at a single hospital.^9^ Furthermore, Tuuli et al reported in a meta‐analysis that SCH was associated with preterm delivery, PROM, and placental abruption.^10^ Meanwhile, our results revealed no significant association between SCH in early pregnancy and HDP, NRFS, FGR, CAM, and PROM.

The development of SCH is considered to result from the rupture of blood vessels due to the abnormal invasion of the trophoblast cells into the endometrium. Such a dysregulation of the trophoblastic invasion may equally be involved in the development of abnormal placental adhesion. On the contrary, Heller et al reported that the size of SCH based on the fraction of gestational sac correlated with the first trimester miscarriage, rather than the presence or absence of SCH.^17^ Thus, the relationship between SCH and pregnancy complications may similarly be related to the location and size of SCH. We aim to determine whether these factors affect the results of pregnancy complications in a subsequent study.

This study has several limitations. First, the contribution of SCH to miscarriage is an important point; however, we did not focus on this subject because most miscarriages are caused by chromosomal abnormalities, and the product of conception/karyotype was not examined. Second, SCH could be formed by bleeding due to vascular breakdown, although it is unclear whether vaginal bleeding is caused by SCH. Third, the presence of SCH after 12 weeks of gestation, in addition to the size and location of SCH, was not evaluated; however, it is known that a significant portion of the SCH during early pregnancy disappears before delivery. However, these results suggest that the presence of SCH in early pregnancy is a risk factor for abnormal placental adhesion, regardless of the presence or absence of SCH after the second trimester. Finally, we could not evaluate some pregnancy complications, including gestational diabetes, placenta abruption, oligohydramnios, and polyhydramnios due to the limited number of cases. Therefore, these issues need to be further evaluated in a future study.

In conclusion, these results suggest that SCH in early pregnancy may contribute to the development of abnormal placental adhesion and placenta previa in pregnancy resulting from FET. Further, it was similarly observed that SCH with vaginal bleeding may be one of the causes of the higher frequency of abnormal placental adhesion. The presence of SCH should be carefully noted, particularly in cases of SCH with vaginal bleeding during early pregnancy after FET.

## CONFLICT OF INTEREST

S. So and N. Murabayashi are affiliated with the laboratory of Tawara IVF clinic, which funded the study. All other authors declare that they have no conflict of interest.

## ETHICAL APPROVAL

The protocol for this study was approved by the Institutional Review Board (IRB) of Tawara IVF clinic.

## HUMAN RIGHTS STATEMENTS AND INFORMED CONSENT

All procedures followed were in accordance with the ethical standards of the responsible committee on human experimentation (institutional and national) and with the Helsinki Declaration of 1964 and its later amendments. Informed consent was obtained from all patients for being included in the study.

## Supporting information

Table S1

## References

[1] Ishihara O , Jwa SC , Kuwahara A , et al. Assisted reproductive technology in Japan: a summary report for 2016 by the Ethics Committee of the Japan Society of Obstetrics and Gynecology. Reprod Med Biol. 2018;18:7‐16. 30655717 10.1002/rmb2.12258 PMC6332769

[2] Takeshima K , Jwa SC , Saito H , et al. Impact of single embryo transfer policy on perinatal outcomes in fresh and frozen cycles‐analysis of the Japanese Assisted Reproduction Technology registry between 2007 and 2012. Fertil Steril. 2016;105(2):337‐346.e3. 26518122 10.1016/j.fertnstert.2015.10.002

[3] Griesinger G , von Otte S , Schroer A , et al. Elective cryopreservation of all pronuclear oocytes after GnRH agonist triggering of final oocyte maturation in patients at risk of developing OHSS: a prospective, observational proof‐of‐concept study. Hum Reprod. 2007;22:1348‐1352. 17303632 10.1093/humrep/dem006

[4] Griesinger G , Schultz L , Bauer T , Broessner A , Frambach T , Kissler S . Ovarian hyperstimulation syndrome prevention by gonadotropin‐releasing hormone agonist triggering of final oocyte maturation in a gonadotropin‐releasing hormone antagonist protocol in combination with a "freeze‐all" strategy: a prospective multicentric study. Fertil Steril. 2011;95(2029–33):2033.e1. 10.1016/j.fertnstert.2011.01.163 21371705

[5] Ishihara O , Araki R , Kuwahara A , Itakura A , Saito H , Adamson GD . Impact of frozen‐thawed single‐blastocyst transfer on maternal and neonatal outcome: an analysis of 277,042 single‐embryo transfer cycles from 2008 to 2010 in Japan. [published correction appears in Fertil Steril. 2014 Apr; 101(4):1200] Fertil Steril. 2014;101:128‐133. 24268706 10.1016/j.fertnstert.2013.09.025

[6] Maheshwari A , Pandey S , Amalraj Raja E , Shetty A , Hamilton M , Bhattacharya S . Is frozen embryo transfer better for mothers and babies? Can cumulative meta‐analysis provide a definitive answer? Hum Reprod Update. 2018;24:35‐58. 29155965 10.1093/humupd/dmx031

[7] Saito K , Kuwahara A , Ishikawa T , et al. Endometrial preparation methods for frozen‐thawed embryo transfer are associated with altered risks of hypertensive disorders of pregnancy, placenta accreta, and gestational diabetes mellitus. Hum Reprod. 2019;34:1567‐1575. 31299081 10.1093/humrep/dez079

[8] Pearlstone M , Baxi L . Subchorionic hematoma: a review. Obstet Gynecol Surv. 1993;48:65‐68. 8437776

[9] Nagy S , Bush M , Stone J , Lapinski R , Gardó S . A subchorialis és retroplacentaris haematomák szülészeti következményei. [Clinical significance of subchorionic and retroplacental hematomas detected in the first trimester of pregnancy] Orv Hetil. 2005;146:2157‐2161. 16315997

[10] Tuuli MG , Norman SM , Odibo AO , Macones GA , Cahill AG . Perinatal outcomes in women with subchorionic hematoma: a systematic review and meta‐analysis. Obstet Gynecol. 2011;117:1205‐1212. 21508763 10.1097/AOG.0b013e31821568de

[11] Asato K , Mekaru K , Heshiki C , et al. Subchorionic hematoma occurs more frequently in in vitro fertilization pregnancy. Eur J Obstet Gynecol Reprod Biol. 2014;181:41‐44. 25126980 10.1016/j.ejogrb.2014.07.014

[12] Xiang L , Wei Z , Cao Y . Symptoms of an intrauterine hematoma associated with pregnancy complications: a systematic review. PLoS One. 2014;9:e111676. 25369062 10.1371/journal.pone.0111676 PMC4219764

[13] Anestad G , Lunde O , Moen M , Dalaker K . Infertility and chlamydial infection. Fertil Steril. 1987;48:787‐790. 3666181 10.1016/s0015-0282(16)59531-4

[14] Owen CA , Campbell EJ . The cell biology of leukocyte‐mediated proteolysis. J Leukoc Biol. 1999;65:137‐150. 10088596 10.1002/jlb.65.2.137

[15] Jia RZ , Rui C , Li JY , Cui XW , Wang X . CDX1 restricts the invasion of HTR‐8/SVneo trophoblast cells by inhibiting MMP‐9 expression. Placenta. 2014;35:450‐454. 24836459 10.1016/j.placenta.2014.04.011

[16] Truong A , Sayago MM , Kutteh WH , Ke RW . Subchorionic hematomas are increased in early pregnancy in women taking low‐dose aspirin. Fertil Steril. 2016;105:1241‐1246. 26820772 10.1016/j.fertnstert.2016.01.009

[17] Heller HT , Asch EA , Durfee SM , et al. Subchorionic hematoma: correlation of grading techniques with first‐trimester pregnancy outcome. J Ultrasound Med. 2018;37:1725‐1732. 29341210 10.1002/jum.14524

