# Supplementary material for: Impact of subchorionic hematoma in early pregnancy on obstetric complications: A retrospective cohort study in women who had live births after frozen‐thawed embryo transfer
Source: Reprod Med Biol. 2020 Aug 5;19(4):398–403. doi: 10.1002/rmb2.12343 (PMC7542017; doi:10.1002/rmb2.12343)
Supplement: Supplementary file 1 — Table S1 [file RMB2-19-398-s001.docx]

Supplemental Table 1 Surgery of endometrial polyps before embryo transfer cycle

| Surgery | Non-SCH group | SCH group | P value |
| --- | --- | --- | --- |
| D&C | 17.0% | 31.3% | 0.1260 |
| TCR | 37.7% | 43.8% | 0.5834 |
| Observation | 45.3% | 25.0% | 0.0615 |

SCH, subchorionic hematoma; D&C, dilatation and curettage; TCR, transcervical resection
